# The Role of Graphic Design Semiotics in Environmental Awareness Campaigns

**DOI:** 10.3390/ijerph20054299

**Published:** 2023-02-28

**Authors:** Marc Vallverdu-Gordi, Estela Marine-Roig

**Affiliations:** 1 Economics and Business Studies, Open University of Catalonia, 08035 Barcelona, Spain; 2 Economics and Business Department, University of Lleida, 25001 Lleida, Spain

**Keywords:** semiotics, graphic design, social marketing, environmental awareness campaign, tourist behaviour, sustainable destination, structural equation modelling, natural park, Catalan Pyrenees, France

## Abstract

This work aims to explore the usefulness of graphic design in awareness campaigns promoting sustainable tourist destinations and to identify their contribution to the success of the campaigns in terms of their generating increased protection of the natural and socioeconomic resources of the destination. The study applies semiotics to the field of social marketing to build a conceptual model that relates the graphic design of a campaign to public environmental awareness, and to the destination’s preservation. In order to test the conceptual model, the campaign “Que la montagne est belle!” of the “Parc Naturel Régional des Pyrénées catalanes” in the French Pyrenees is taken as a case study for analysis, as it aims to preserve the park’s natural environment and pastoral activities. The data are analysed using the partial least squares structural equation modelling technique (PLS-SEM), and the results are studied for different segments of the sample. The findings show that the graphic design semiotics influence public environmental awareness and destination preservation by generating in the audience a sensitive, emotional, and cognitive reaction towards the campaign. This innovative framework on graphic design can be adapted to other branding or marketing campaigns to improve destination images.

## 1. Introduction

Tourist activities are inseparable from the surrounding environment. In this way, it is necessary to make tourists, residents, and stakeholders aware of making the tourism industry sustainable by adopting sustainable practices [1]. The sustainability of local resources is a key element to improve the image and competitiveness of tourist destinations [2]. The current demand for new tourist models turns protected natural spaces into successful tourist destinations that offer tourists unique and authentic experiences for an escape from their routines. However, at the same time, these spaces have much stricter requirements for their preservation than conventional tourist destinations do. Therefore, it is necessary that these spaces be transformed into sustainable tourist destinations where tourism is in balance with environmental protection, together with the preservation of the cultural and socio-economic resources of the populations that inhabit the area [3].

The destination’s ability to attract tourists depends on the good condition of these resources, and the economic, social, and environmental success of the destination could be disrupted if its resources were degraded as a result of damaging human behaviour or poor management by the administration [4]. This is why it is recommended that sustainable tourist destinations provide access to quality information about the territory’s resources and carry out education and awareness campaigns aimed at influencing tourists, residents, and tourist operators to reduce their negative impacts on the destination’s resources. This translates into greater preservation of such destinations and a more equitable balance between tourism development and environmental protection [3].

These awareness campaigns are communication campaigns, which, like destination promotion campaigns, must incorporate and make good use of information and communication technologies (ICT) and the tools and knowledge that allow these technologies to be applied correctly if they are to be effective and competitive in the current technological and socio-cultural context that determines contemporary tourist models [5]. One such field of knowledge is graphic design, which is indispensable in any communication material associated with a campaign of this nature as it is concerned with attracting the public’s attention and ensuring their understanding of the transmitted messages [6].

Understanding the contribution of the campaigns’ graphic design to raising visitors’ and residents’ awareness of the importance of preserving the destination’s resources allow us to identify specific design actions that increase the effectiveness in achieving the campaign’s objectives. Therefore, this work will focus on graphic design to define the best practices that lead to the effective preservation of the resources of a protected natural space as result of visitor and inhabitant education. The study will be carried out from the perspective of semiotics, a discipline that investigates the perceived meaning of the signs and messages sent to the users of a destination through an awareness campaign and the effects of these perceptions on their behaviours.

A conceptual model will be created to identify causal relationships between the graphic design of awareness campaigns, the behaviour of the campaign audience and the protection of tourism destination resources. This model will be empirically validated through the application of the structural equation modelling (SEM) analysis technique in the data of a real case of protected space, that of the campaign “Que la montagne est belle!” (How beautiful the mountain is!) (QLMEB) of the “Parc Naturel Régional des Pyrénées catalanes” (Regional Natural Park of the Catalan Pyrenees) (PNRPC) [7]. This is an exemplary case in the field of sustainable tourist destinations, since the figure of special protection of regional natural parks in France is a status that incorporates and protects the coexistence of natural, cultural, and socioeconomic resources, while revitalizing tourism [8].

The analysis of the case study of the QLMEB campaign makes it possible to validate the causal relationships of the conceptual model by answering: (1) whether the design of the QLMEB campaign, from graphic and conceptual points of view, has been capable of impacting and attracting the attention of PNRPC users (visitors and residents) at a visual, cognitive and emotional level; (2) whether the design of the QLMEB campaign has contributed to sensitizing users to the protection of the natural environment and pastoral activities of the park; and (3) whether the design of the QLMEB campaign has contributed to protecting the natural environment and pastoral activities of the PNRPC by deterring behaviours that put these resources at risk.

The role of graphic design in the success of awareness campaigns is an under explored area of knowledge, so there is no literature on this subject in connection to the preservation of natural resources or to the sustainability of tourist destinations. This gap of knowledge means that the present study represents an opportunity for an initial exploration of the topic, providing basic knowledge for future studies. This study also involves the discovery and exploration of new methodological applications, such as the semiotic perspective and the SEM analysis, to studies of public awareness campaigns, though both have been used in research on commercial advertising campaigns and customer satisfaction [9,10].

Therefore, this study proposes and justifies a conceptual model that relates four constructs: the graphic design semiotics; sensory, affective, and cognitive experiences; environmental awareness of the audience; and preservation of the destination. Next, it includes a summary of the literature that supports the model. Given that the case study is a common way of testing conceptual models in the field of tourism [11], the new model is applied to the QLMEB campaign promoted by the PNRPC park, and the relationships between constructs are analysed through the partial least squares structural equation modelling (PLS-SEM) technique.

## 2. Theoretical Background

Tourism is an important driving force for the economic and social development of destinations in both developed and developing countries. However, tourism can also be the cause of significant damage and disturbances to the environment and the socio-economic structure of a territory through the overconsumption of natural resources and worsened living conditions of local inhabitants [12,13].

Sustainable tourism is presented as a solution to the externalities of the tourism sector, defined as that model of tourism that “ensures the long-term protection and preservation of natural, cultural and social resources, and contributes positively and equitably to the economic development and well-being of the individuals” [3] in protected areas. In the context of sustainable tourism, the preservation of the destination is understood as maintaining the economic and social systems essential to the inhabitants’ quality of life, the area’s cultural integrity, the ecological processes fundamental to the correct functioning of the ecosystems, and the biological diversity of the territory where tourism is developed [14].

The PNRPC is a sustainable tourist destination, since its statutes report that it seeks to preserve and protect the landscape, natural heritage and cultural heritage while contributing to economic, social, and cultural development and improving the quality of life of its inhabitants [7]. In this context, the QLMEB campaign is established as a communication tool to fulfil the preservation objectives that derive from the exercise of a sustainable tourism model, which has been subscribed to by the municipalities that make up the PNRPC.

### 2.1. Graphic Design Semiotics Applied to the Analysis of Communication in Tourism

The field of semiotics studies the meaning and interpretation given to signs—understanding them as the visual and/or linguistic representations used for human communication—and tries to understand the human behaviour that results from sign interpretation. The signs of a communication product, i.e., an advertisement or the packaging of a product, are made up of verbal elements (letters, words and oral and written messages) and non-verbal elements (colours, shapes and typographic styles) [10,15]. During the conceptual and graphic design process of the campaign or communication product, these elements are deliberately combined to form signs that impact the audience’s experience. The meanings and interpretations given to signs vary greatly depending on the cultural and historical context of where the information is shared [15,16].

Catellani [17] highlights the importance of environmental communication campaigns that combine text and images from a semiotic perspective. Therefore, graphic design, defined as the visual communication resulting from the combination and organization of images and words to convey ideas [18], from the point of view of semiotics, is understood as a tool that can create signs and produce reactions to these signs that result from the public’s interpretations, while making use of graphic elements, e.g., colours, shapes, typography, etc. That is, applied semiotics uses knowledge about signs to achieve aims such as the modification of consumer behaviour [19]. Moreover, in their typical touristic behaviour, tourists are the agents of semiotics: all over the world they are engaged in semiotic projects, reading landscapes and cultures as sign systems [16]. Thus, semiotic analysis is useful for researchers in the field of tourism to understand and predict the behaviour of tourists vis-à-vis a communication product by studying the visual and linguistic signs that the product uses and that result from a process of graphic design [15]. In this vein, infographic messages composed of images and text are more persuasive than communications that rely solely on text or illustrations, and they have proven useful in communicating environmental issues [20].

Consequently, the application of the semiotics perspective is very suitable for the case of the QLMEB communication campaign, since it allows us to measure its success by analysing how the signs used in the graphic composition of the campaign affect the behaviour of tourists of the PNRPC and its inhabitants, while expecting to identify a positive change in behaviour that preserves the Park’s environment and a reconciliation of pastoral and tourist activities.

### 2.2. Awareness Campaign and Social Marketing from the Perspective of Semiotics

From the perspective of semiotics, an awareness campaign is considered a typology of communication or a marketing campaign called social advertising or social marketing by various authors [21,22]. It is defined as a communication practice aimed at raising awareness, meaning to motivate a behavioural change in the public that, unlike usual marketing campaigns, is not aimed at consumption or loyalty to a product or brand, but at positive environmental and/or social behaviours by making certain social and/or environmental problems known to the public [5,23]. Then, given the conceptual link between awareness campaigns and marketing campaigns, the evaluation and analysis of an awareness campaign can be carried out with the same tools as any other marketing or branding campaign: surveys, focus groups, and neuromarketing [5].

This is why the methodology of analysis and interpretation of QLMEB will be able to take studies of semiotics and graphic design in branding, such as Shukla et al. [10], as a reference, which investigates the effectiveness of a branding campaign design through the concept of branding experience.

### 2.3. Awareness and Awareness Campaign Experience

Branding experience refers to the set of impacts subjectively generated on an individual by the stimuli perceived from a brand promotion campaign [24]. These stimuli are mainly derived from the design of a campaign, although there are also contextual social and environmental factors that affect the public when exposed to a campaign [9]. Other authors [25] have recommended awareness campaigns targeting tourists and residents to improve their affinity to a protected area because respondents with low national-park affinity had more negative attitudes towards natural resource conservation. Taking the link between the concepts of awareness campaigns and branding–marketing campaigns [5,23], the concept of branding experience can be adapted to the concept of awareness campaigning. In this way, the awareness campaign experience can be defined as the set of impacts generated subjectively on an individual by the stimuli perceived from an awareness campaign, especially via its design.

As a result of this new concept, and adapting the model of Shukla et al. [10], the present study will seek to learn about the awareness campaign experience, i.e., the experience perceived by PNRPC users when in contact with the QLMEB’s communicative products, and will seek to determine the relationship between the awareness campaign experience and the degree of awareness, since this experience in particular will affect the campaign’s ability to motivate positive changes in the behaviour of its audience.

Previous studies [24,26] have segmented the experience of a branding campaign into sensory, cognitive, affective, and behavioural dimensions. Thanks to the conceptual links presented above, the same segmentation will be considered for the awareness campaign experience concept. In this way, the segmentation of the experience of an awareness campaign presents the following dimensions: (1) the sensory experience, which refers to the perceptions produced by the awareness campaign captured through the senses—mainly visual perceptions; (2) the cognitive experience, which refers to the knowledge transmitted and the reflections produced by the awareness campaign; (3) the emotional–affective experience, referring to the feelings and emotions evoked by the awareness campaign; and finally (4) the behavioural experience, which refers to the actions and behaviours inspired by the awareness campaign. 

In this study, the concept of behavioural experience [24], which refers to the positive behavioural changes in the campaign audience, will be integrated within the broader concept of awareness, which incorporates not only behavioural changes, but also changes in thinking and unmaterialized intentions to change behaviours. This is because the concept of awareness, within the framework of social marketing and awareness campaigns, is defined as a set of actions intended not only to cause a change in attitude and behaviour of the public towards a specific problem, but also to generate knowledge and changes in thinking [5]. The behavioural dimension of the experience, in the case of QLMEB, refers to the changes in behaviour and the actions of protection towards the natural environment and pastoral activities of the PNRPC, motivated by the campaign, and which are the result and the objective of the campaign.

### 2.4. Conceptual Model Approach

The design of an advertising and communication campaign, as well as the design of a product and its packaging, made up in all cases by colours, shapes, logos, images, and in some cases even smells and music, represent a set of inputs or stimuli for the audience’s senses that determine their sensory experience when coming into contact with the campaign or product [27]. In this way, through the graphic design of the campaign, those responsible for the campaign can control the sensory experience of the public and, subsequently, their behaviour in relation to the advertised product, or destination in the case of QLMEB.

In advertising and communication campaigns for products, services, or brands (marketing and branding), any stimulus has an effect on the public’s emotions, and the type of stimulus conditions the type of emotional response [9,24,28]. The stimuli correspond to contextual social and environmental factors and, more importantly for the focus of this study, the design characteristics of the campaign [9]. In this way, for example, many tourist destinations use advertising design elements (images, effects, music...) to generate positive emotional responses in the public that arouse their interest in visiting the destination [29]. Thus, an awareness campaign or social advertisement, being a type of marketing campaign, will also be able to generate emotional responses in the public depending on its graphic design stimulus [30]. 

Furthermore, the design of an advertising campaign, among other factors, stimulates the minds of the public [24,27], as the different elements of the design are processed as information, interpreted, used to produce new thoughts and subsequently memorized or forgotten consciously or unconsciously [9,31]. This means that the graphic design of a campaign is a stimulus that generates a cognitive experience in the audience, as it is information that is processed by this audience and that generates new thoughts and knowledge.

Since the visual sense has the greatest influence on consumer decision making [27], and the graphic design production process operates through a particular semiotic structure [32], it is proposed that the graphic design of the campaign determines the audience’s experience in the sensory, emotional/affective and cognitive dimensions.

**H1.**  *The graphic design semiotics of the campaign have a positive impact on the sensory, emotional, and cognitive experience of the audience.*

In a context of sustainable development, the design of public awareness campaigns can be focused on promoting actions that preserve the environment and positive changes in behaviour and awareness of ecological issues [5]. Environmental communication campaigns can be analysed from a semiotic standpoint [17]. Through design semiotics, infographics represent an effective way to convey topics, such as environmental issues, to the target audience [20].

**H2.**  *The graphic design semiotics of the campaign have a positive impact on the preservation of the destination.*

The experience of a marketing campaign in the public can result in changes in behaviour towards the advertised products. Khandelwal et al. [27] focus on the potential of sensory and cognitive experiences to generate behavioural changes in consumers, while Mostafa et al. [9] focus on emotional and cognitive experiences. Given that awareness campaigns represent a type of marketing campaign known as social marketing, the studies presented suggest that the sensory, emotional, and cognitive experiences resulting from an awareness campaign have the ability to raise public awareness and cause changes in thinking and behaviour in an audience.

Moreover, Boraswska [5] presents several examples of successful public awareness campaigns, and, like other authors [21,33,34,35], indicates that well-planned awareness campaigns are effective communication tools for raising awareness among the population, generating positive behavioural changes by making the public aware of environmental issues and needs, and consequently protecting natural environments and promoting sustainable development. Moreover, previous research [25,33] has indicated the potential of awareness campaigns for successfully raising tourists’ awareness and preserving destinations. For the resulting good intentions of tourists to be effectively translated into a change in behaviour, the campaign needs to convey clear information about what, why, and how individuals can make a difference [33], i.e., tourists must be well informed, and the proposed alternatives or changes must be acceptable and convenient for tourists. According to Arnberger et al. [25], environmental education is a primary responsibility of a national park, and rules of visitor behaviour are necessary even to provide a positive visitor experience.

In this way, the aforementioned authors allow us to suggest (1) that the QLMEB campaign and the set of experiences generated in the public by it can positively impact the changes in the public’s thinking and behaviour towards the natural environment and pastoral activities of the PNRPC, and (2) that these changes can be translated into improved protection of these resources. Accordingly, the following hypotheses are defined:

**H3.**  *The different types of experience (sensory, emotional, and cognitive) have a positive impact on the public awareness generated by the campaign.*

**H4.**  *The audience awareness has a positive impact on the actions taken to preserve the natural and socio-cultural environment of the destination.*

**Figure 1:**
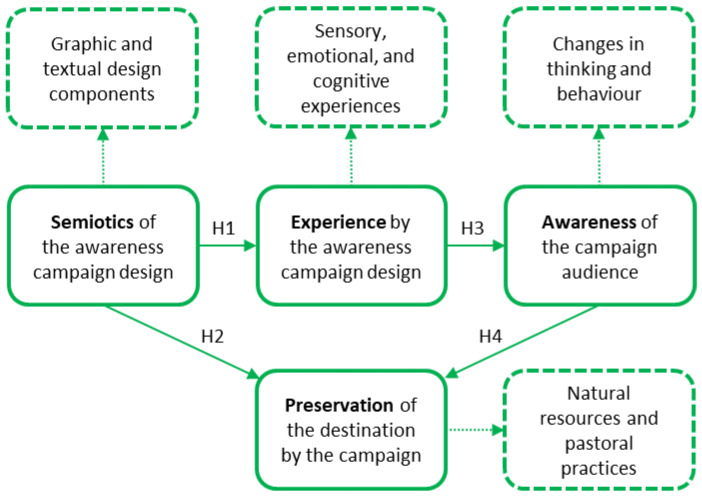
Conceptual model composed of four constructs.

Based on the four hypotheses presented (H1, H2, H3, and H4), Figure 1 shows a conceptual model that incorporates the four constructs previously defined that represent the awareness campaign: the graphic design semiotics of the campaign; the sensory, emotional and cognitive experience of the campaign; the awareness of the public (changes in behaviour and thinking); and the preservation of the destination. The semiotics of the campaign include textual components (written messages) and graphics (colours, shapes, typographies and styles) according to the differentiation proposed by several authors between “verbal” and “non-verbal” semiotic elements [10,15]. The awareness campaign experience concept includes four dimensions: sensory experience, affective experience, cognitive experience, and behavioural experience [24]. However, the fourth dimension is integrated within the awareness construct in relation to the preservation construct, which incorporates the changes in the public’s thinking and behaviour produced by the campaign [5].

### 2.5. Summary of Related Literature

Although, to the best of our knowledge, there is no literature directly related to the conceptual model proposed in this study, Table 1 contains a sample of the publications that address aspects related to graphic design, semiotics, branding, environmental awareness campaigns, and sustainable tourism.

## 3. Materials and Methods

The validation of the conceptual model has been carried out through the case study of the QLMEB of PNRPC awareness campaign. The PNRPC is one of the 58 French Regional Natural Parks (PNR) and is located in the Occitan region of the Eastern Pyrenees department [37]. The public figure of the PNR is regulated at the state level, but it is voluntarily instituted by a local initiative, a group of cooperating municipalities that accept statutes aimed at protecting the environment and promoting the economic, social and touristic development of the territory [8]. The PNRs, including the PNRPC, meet the definition of sustainable tourist destinations because they are territories that take into account the economic, social, and environmental impacts of tourist activities to ensure the long-term preservation of natural, social, and cultural resources, and they contribute to the economic development and well-being of tourists and inhabitants [3,4].

**Figure 2:**
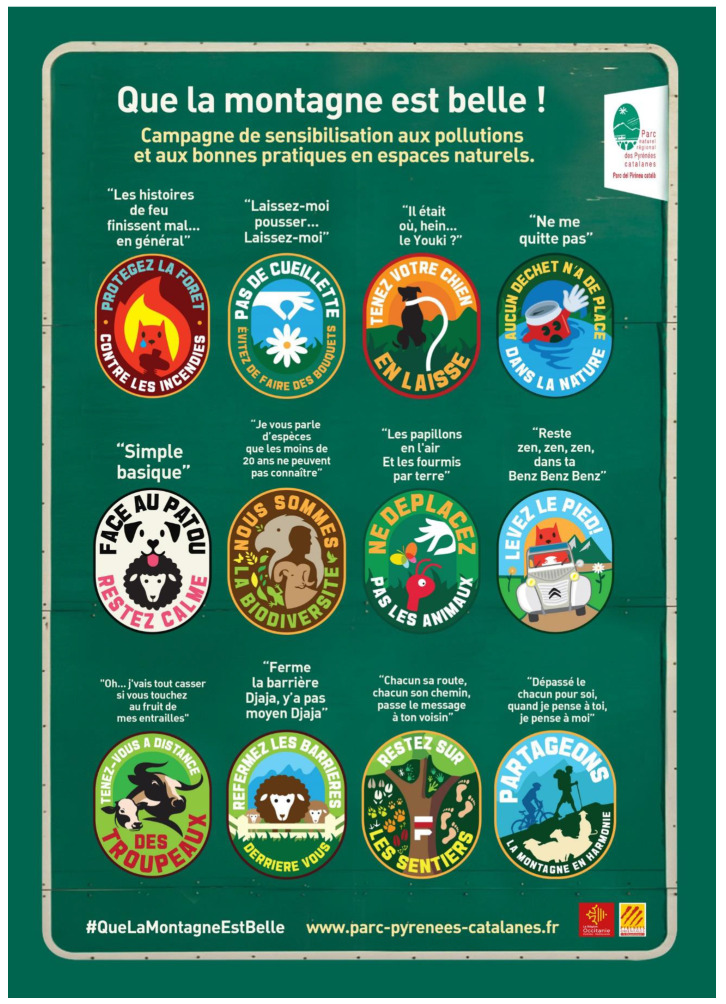
Awareness campaign flyer shared by the Park administration [38].

The QLMEB awareness campaign of the PNRPC started in May 2021 and responds to the objectives of the statutes of the PNRPC [7], which are to raise awareness among tourists and residents about the preservation of the natural and socio-cultural resources of the park, with an especial focus on natural resources and pastoral activities, and to bring about reconciliation between tourist activities, pastoral practices and the protection of the natural environment [38]. The campaign includes 12 messages aimed at solving 12 corresponding problems related to tourist activities in the PNRPC. The awareness campaign has been designed to attract the attention of tourists through two innovative features: (1) a graphic design that presents simple and intuitive symbols in the form of collectable badges, and in a style reminiscent of old road signs; and (2) messages presented through verses from songs known at the French national level that refer to each of the tourist problems discussed (Figure 2).

### 3.1. Survey Instrument and Data Collection

In order to validate the conceptual model and the four hypotheses that make it up by either corroborating or discarding the correlations between the pairs of constructs, quantitative research methods were applied to the collection and analysis of data. The model’s hypotheses were statistically validated through the completion of a survey by visitors and residents of the PNRPC with questions that corresponded to the conceptual model, measured with a 5-point Likert scale ranging from 1 = “do not agree at all” to 5 = “strongly agree”. The survey went through several stages of reducing the number of questions and simplifying them during the validation process with the PNRPC administration. This was carried out to promote a high response rate to the survey and thus reduce the dropout rate. Finally, the survey only includes the minimum questions needed for preliminary results on the QLMEB awareness campaign. The survey is divided into three sections: The first has a total of five mostly demographic questions (language, age, gender, resident/tourist, prior knowledge of the campaign) intended to establish the profile of the public. The second and third sections contain a total of 10 questions that derive from the constructs of the conceptual model and that aim to obtain the information necessary to validate the model through statistical analysis. The second section contains five questions relating to the audience’s sensory, emotional, and cognitive experience of the campaign. The third section contains five questions relating to the design and characteristics of the campaign’s semiotics. Each of the 10 survey questions referring to the four constructs of the conceptual model (Figure 1) were considered observable variables, whereas the four constructs of the model were considered latent variables that condition the observable variables. The awareness and preservation constructs are made up of a single-item variable because the observable variable sufficiently represents the latent variable. Table 2 presents the latent variables and the observable variables grouped according to the causal relationships established in the conceptual model and using the assigned nomenclature.

The survey was disseminated electronically through the PNRPC’s social networks (Facebook and Instagram), in the Park’s monthly newsletter (MailChimp), and among the Park agents’ personal contacts. It was also disseminated in Facebook groups related to high mountain leisure activities, tourism, and other matters of local interest in which inhabitants of the territory of the PNRPC and the department of the Eastern Pyrenees participate. In this way, we sought to reach both inhabitants and tourists of the territory. Table 3 presents the descriptive analysis of the data collected through the survey, which received 149 valid responses. The data indicate that approximately: (a) two thirds of the respondents are residents of the park and its surroundings, while one-third are tourists; and (b) the majority of respondents are between 35 and 60 years old and female.

### 3.2. Analytical Procedures

The analysis of the data obtained in the survey to validate the hypotheses of the conceptual model was carried out through the structural equation modelling (SEM) technique. SEM is a multivariate quantitative statistical analysis technique aimed at identifying and quantifying the direct and indirect causal correlations between the observable variables and latent variables of a model [39]. The model, made up of the variables and the correlations between them, can be represented graphically in a diagram. The SEM makes it possible to estimate the latent variables based on the correlations with the rest of the variables in the model, understanding the latent variables as those that cannot be measured directly due to their complexity and that affect or are affected by the other variables [39].

There are various modes of SEM that adapt to the different needs and structural and methodological limitations of the studies and the data available. In order to find out the type of SEM that best suits the specificities of the data collected for the analysis of the conceptual model created, a normality test was performed on the 10 observable variables of the model. The results of the two normality tests applied to the data, namely Shapiro–Wilk and Kolmogorov–Smirnov, indicate that none of the 10 variables follows a normal distribution [40], obtaining p-values lower than 0.05 for all statistics, which meant that the hypothesis of normality had to be rejected. With this information, it was decided that the SEM technique of partial least squares (PLS-SEM) should be applied, since its requirements are adapted to those of the characteristics of the data set of each of the observable variables obtained through the survey [41]: (a) it does not require very large samples, (b) it does not require the assumption of normal distribution of the data, and (c) it allows the calculation of correlations despite the lack of some data. In this vein, another major advantage is that PLS-SEM allows the analysis of single-item constructs, because, as stated by Hair et al. [42], “it permits the unrestricted use of single-item and formative measures” (p. 7). Moreover, according to Garson [43], “single-item variables may cause identification and convergence problems in covariance-based SEM, but this is not a problem in PLS-SEM” (p. 31).

Since both SEM and PLS-SEM support the reflective and formative models, scholars agree that the selection of the appropriate technique depends on the nature of the indicators. Briefly, the formative model is chosen when the latent variable is composed of the measurements and the reflective model is chosen when the measurements are representative of the latent variable [43]. In addition, PLS-SEM is a model indicated for exploratory studies of new models where there is little prior knowledge about the causal relationships among the data, which is the case of the present study. SmartPLS 3 software was used to apply PLS-SEM to the data, while IBM SPSS software was used for all other statistical treatments. SmartPLS is a scientifically grounded software used in most PLS-SEM studies [41].

In order to determine if differences between the results of different demographic profiles of the public exist and they consist of, the present study chose to segment the data based on the tourist-inhabitant, gender and age (up to 60 years old and over 60 years old) demographic variables, applying the PLS-SEM analysis separately in each of these segments. Then, the influence of these variables was determined by identifying if there are differences between the validation and the weight of the correlations between latent variables for each of the segments and in relation to the results obtained from the global set of data. Applying PLS-SEM to sample segments is possible because it is an analysis technique indicated for small sample sizes [41].

### 3.3. Assessing Overall, Measurement, and Structural Models

The preliminary step was to estimate the sample size needed to be able to apply the PLS-SEM analysis technique to all the data and to each of the sample segments. Two conventional methods of sample size estimation have been used: the 10-times rule and the Minimum R-squared methods [44]. The results of the PLS path modelling can be evaluated globally (overall model) and locally (measurement and structural models) [45].

Evaluating the results of PLS-SEM involves examining both measurement and structural models [42]. In this case study, the measurement model was reflective because the indicators could be considered representative samples within the constructs. In the reflective measurement model, the basic measures to assess the reliability of the results are: (a) reflective indicator loadings; (b) internal consistency reliability; (c) the construct’s convergent validity; and (d) discriminant validity. In the structural model, the basic measures are: (a) collinearity statistics; (b) path coefficients; (c) coefficient of determination (R-square); (d) effect size (f-square); and (e) predictive relevance (Q-square).

## 4. Results and Discussion

**Figure 3:**
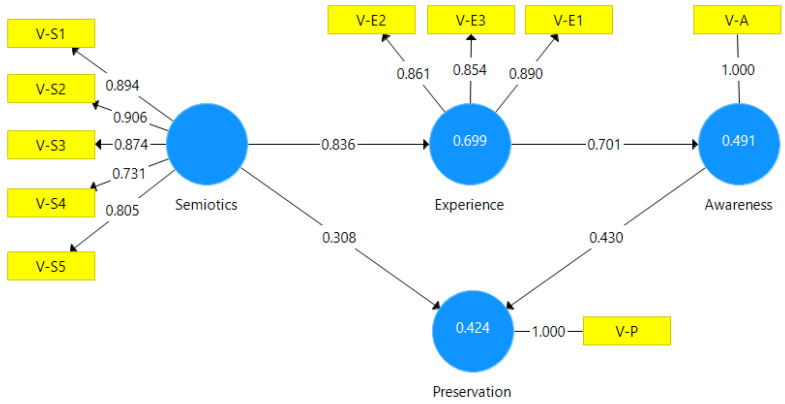
Partial least squares (PLS) regression analysis.

For the PLS-SEM analysis, Figure 3 shows the conceptual model of Figure 1 with the latent and observable variables of Table 2. Figure 3 also includes the results of running the PLS algorithm (outer loadings, path coefficients, and R-squared) with the aim of testing the validity of the model hypotheses.

### 4.1. Minimum Sample Size and Global Model Fit Assessment

According to the “10 times” rule, the minimum sample size has to be ten times the number of paths entering the dependent latent variable with the most latent variables impacting it. In this case, the Preservation construct has two incoming paths, so the minimum size is 20 [46]. Considering the “Minimum R-squared” method, given that the maximum number of arrows pointing to a latent variable is two and the minimum R-squared of the model is 0.424, the minimum size that fits in the table [44] is 39. Therefore, the sample has the minimum size required for a PLS-SEM analysis, even when segmented through demographic variables.

The global goodness-of-fit of the model represents the starting point for the evaluation of the model [45]. The standardized root mean square residual (SRMR) is a measure of approximate fit of the researcher’s overall model. SRMR measures the difference between the observed correlation matrix and the model’s implicit correlation matrix. The SRMR values (saturated model: 0.073; estimated model: 0.075) indicate that there is a relatively good fit (cut-off close to 0.08) between the conceptual model and the observed data [47].

### 4.2. Reflective Measurement Model Assessment

Reflective indicator loadings: All the values of the loadings (Figure 3) are greater than 0.708 [42], which demonstrates an acceptable reliability of the item, because the construct explains more than 50% of the indicator’s variance. Generally, the larger the loadings, the stronger and more reliable the measurement model is.

Internal consistency reliability. The two main criteria to determine this reliability are Cronbach’s alpha representing the lower bound, and Composite reliability representing the upper bound (Table 4). The true construct reliability is usually considered to be between these two extreme values [42]. Both measures vary between 0 and 1 and a higher value represents greater reliability. Therefore, the values of the semiotics and experience constructs are satisfactory [48].

Construct’s convergent validity. Convergent validity is the extent to which the construct correlates positively to explain the variance of its items. The metric used to assess validity is the average variance extracted (AVE). A value greater than 0.50 is considered acceptable [46] because the construct, on average, explains at least 50% of the variance of its indicators. Table 4 shows the AVE values greater than 0.50.

Discriminant validity: Discriminant validity is the extent to which a construct is empirically different from other constructs in the model. There are two traditional criteria for evaluating the uniqueness of constructs: (1) The Fornell–Larcker criterion compares the correlations of the latent variables with the square root of the AVE of the construct, which must be higher than its correlations with any other construct [49]. Applying the criterion, it turns out that the values of the diagonal are higher than the other values of the rows and columns, indicating that the constructs represent unique concepts. (2) Similarly, the outer loading of an indicator on the associated constructs must be greater than any of its cross-loadings on another construct. Table 5 displays that the values of the diagonal are higher than the values of the respective columns, showing that the discriminant validity is well established.

### 4.3. Structural Model Assessment

Collinearity statistics. The first step in evaluating structural relationships is to check for collinearity to ensure that it does not bias the regression results. Multicollinearity exists when two or more independent variables are highly intercorrelated, which prevents the researcher from assessing the relative importance of one independent variable versus the other. The variance inflation factor (VIF) is the measure commonly used to assess collinearity. Values below the threshold of 5 indicate that collinearity is not a critical problem in the structural model, but values below 3 are ideal [50]. In the proposed model, both inner and outer VIF values are less than 5, but there are two values between 3 and 5 (V-S1 = 3.899; V-S2 = 4.405), indicating the possibility of collinearity issues [42].

Path coefficients. The next step is to check the statistical significance and relevance of the path coefficients. Normally, they are included in a range from −1 to +1. A coefficient close to +1 represents a strong relationship between the constructs and is usually statistically significant. Table 6 shows that the strongest relationship is Sem → Pre (0.836) followed by Pre → Awa (0.701), and the most important driver for Preservation is Awa → Pre (0.430) followed by Sem → Pre (0.308). All path coefficients are significant [46]. 

Coefficient of determination: The R-squared coefficient is the most commonly used measure to evaluate the structural model, and it explains the variance in the endogenous variable introduced by the exogenous variable(s). The value ranges from 0 to 1, and a higher score indicates a higher level of predictive accuracy or explanatory power. The values (0.699, 0.491, 0.424) inside the latent endogenous variables in Figure 2 can be considered moderate [42].

Effect size. To assess whether the omitted constructs have a substantive impact on the endogenous latent variables, the change in R square, when omitted from the model, was calculated using the f-square effect size. Two of the resulting values are higher than 0.35 (Sem → Exp: 2.323; Exp → Awa: 0.965), one is higher than 0.15 (Awa → Pre: 0.288), and one is higher than 0.02 (Sem → Pre: 0.117). Therefore, the effect sizes are large, medium, and small, respectively [51]. Some authors consider f-square to be a redundant measure [41].

Predictive relevance. For a specific reflective endogenous construct, the Q-square values larger than zero indicate the path model’s predictive accuracy for a particular dependent construct. The Q-square values in Table 4, higher than 0.25 and 0.50, depict medium and large predictive relevance of the PLS-path model [42].

### 4.4. Discussing Correlations: Full Sample and Demographic Subsamples

Now that it has been verified that all the samples have the minimum size to be analysed by the PLS-SEM technique and that the model in Figure 3 is reliable and valid from the global, measurement, and structural perspectives, the hypotheses proposed in the conceptual model (Figure 1) are discussed below.

Hypothesis 1 (Sem → Exp). The modelling found a significant causal relationship between the semiotics and experience constructs. The two outer loadings related to colours, images and icons (V-S1 and V-S2) have the most influence on the semiotics construct. In the case of experience, the outer loading with the most influence is sensory (V-E1), followed by emotional (V-E2) and cognitive (V-E3). In all cases (Table 5 and Table 6), the strongest path coefficient found in the model is between semiotics and experience. This positive result can be explained by the graphic design chosen for the QLMEB campaign, together with the use of French songs. These tools were used to make the campaign attract its audience’s attention and stimulate their senses. At the same time, these tools created memories and longer-lasting thoughts in the minds of the public by generating reflections on the campaign’s themes and stimulating cognitive experience.

Hypothesis 2 (Sem → Pre). In all cases, the weakest path coefficient in the model is between the semiotics and preservation constructs (Table 6 and Table 7). Modelling even detected an invalid correlation for those over 60 years of age. These results may indicate the need for experience and awareness as mediators between the semiotics of graphic design and the preservation of the park’s natural resources. 

Hypothesis 3 (Exp → Awa). In all cases, the path coefficient between Experience and Awareness is the second strongest in the model (Table 6 and Table 7). The groups that stand out above the whole (0.701) are those up to 60 years old (0.761) and the local inhabitants (0.731). The great influence of experience on awareness may be due to the fact that the definition of awareness includes changes in thinking—cognitive changes—along with changes in behaviour [5]. If the campaign has a great capacity to reflect and generate new thoughts in the audience’s mind, these new thoughts and reflections may replace or modify preconceived ideas or pre-assimilated attitudes.

Hypothesis 4 (Awa → Pre). Finally, a statistically significant causality was observed between the ability to raise public awareness and the preservation of the park’s natural resources and pastoral activities. The path coefficient of the group of inhabitants (0.503) stands out from the rest (Table 7). This greater influence of the campaign on awareness and preservation may be due to the fact that the inhabitants were previously familiar with the problems that the awareness campaign exposes because they live with these problems in their day-to-day life. In many cases, they are affected by them and are therefore more predisposed to change their behaviour to solve them. In other words, the cognitive stimulus of the campaign is capable of generating greater changes in behaviour because the inhabitants are more susceptible to deciding to make these changes. This hypothesis agrees with the differences in attitude towards the environment identified in tourists by some authors, whose research has indicated that individuals are more willing to participate actively in the protection of the environment in the place where they live than when they are travelling and visiting destinations as tourists [36].

In summary, the results of the analysis (Table 7) show that there are few variations by gender in relation to the sample as a whole (Table 6), although the path coefficients for men are slightly higher than those for women. The results also determine that the tourist-inhabitant factor does not influence the correlations between the constructs of the conceptual model in the case of the QLMEB campaign, but the group of inhabitants positively influences the weight of the correlation between experience, awareness, and the park’s protection. From the analysis that was carried out, it can be affirmed that the graphic design semiotics of the QLMEB campaign statistically influenced the improvement of the preservation of the natural environment and pastoral practices of the PNRPC by generating sensorial, emotional, and cognitive experiences that enhanced the awareness of the park in local inhabitants and tourist visitors.

## 5. Conclusions

Considering that no prior studies have dealt with the relationship between graphic design semiotics and the success of awareness campaigns, this study is a pioneer in its research topic in the following ways: It incorporates elements of marketing studies, specifically by adapting the concept of brand experience to awareness campaign experience. It discusses the relationship between graphic design and the success of awareness campaigns within the theoretical framework of semiotics. Moreover, it applies, as an analysis methodology, structural equation modelling (SEM) to a conceptual model that relates the different identified constructs. In these ways, this study represents the starting point for future research on the role of graphic design.

### 5.1. Theoretical Contribution

The present study has made it possible to create a conceptual model that enables the study of, from the point of view of semiotics, the influence of graphic design in an awareness campaign for tourist destinations on the campaign’s success in encouraging respectful and sustainable behaviours among the tourists and inhabitants. The model was able to be tested thanks to its application to the case of the PNRPC, a French sustainable tourist destination that in 2021 developed such a campaign aimed at improving the behaviours of tourists and inhabitants towards the natural resources and the pastoral practices in the park.

For the development of the conceptual model, the theoretical basis of social marketing was used [21,23] and studies on branding and marketing that investigate the effect of the semiotics of advertising campaigns and the packaging of products and brands on the behaviour and purchase intention of users were taken as a reference [9,10]. The resulting conceptual model was the product of identifying a series of causal relationships between the constructs of semiotics, experience towards the campaign, public awareness, and preservation of the natural resources, which were established as hypotheses to be validated in order to authenticate the model. The collection of data, carried out with a survey disseminated online on social networks, made it possible to acquire sufficient data to carry out the process of statistical validation of the hypotheses through multivariate quantitative analysis techniques. Specifically, the PLS-SEM technique was used to demonstrate that:

- The design of the QLMEB campaign was able to impact and attract the attention of visitors and inhabitants of the PNRPC.
- The design of the QLMEB campaign contributed to sensitising visitors and inhabitants of the PNRPC to the protection of natural and socio-economic resources.
- The design of the QLMEB campaign contributed to the protection of the natural environment and the pastoral practices of the PNRPC.

In this way, it can be concluded that the QLMEB campaign’s graphic design contributed positively and widely to its success and achievement of its objectives, that is, to reconcile tourism with pastoral activities and protect the natural environment of the PNRPC.

The analysis has also shown that an awareness campaign’s use of original graphic design strategies and familiar elements to attract attention has significant potential to generate changes in the audience through cognitive experience. The differences between the degrees of correlation of the different profiles indicate that inhabitants could be more prone than tourists to assume changes in thinking and behaviour favourable to protecting the destination. It is important to note that the correlation between graphic design and preservation of the destination is the weakest in both overall and segmented data, which suggests the need for an environmental awareness campaign to obtain better results.

### 5.2. Managerial Implications

The research carried out makes it possible to verify the importance of planning and of correctly executing the design stage to achieving success in an awareness campaign. The example of QLMEB shows that visually attractive graphic designs that facilitate the transmission and understanding of information with the dissemination of clear and concise messages are more capable of generating ideas and reflections responsible for the changes in thinking and behaviour necessary for the preservation of the natural and socio-cultural environment of a tourist destination. It also shows that the incorporation of familiar elements, such as popular songs in the case of QLMEB, and original and creative elements, such as the design of the QLMEB campaign as collectable badges/emblems, stimulates a cognitive response in the audience, generating curiosity, lasting memories about the messages, a desire to know more and to consume the communicative products of the campaign. This results in an increase in the public’s predisposition to carry out the actions proposed by the campaign during their visit to the tourist destination.

Therefore, managers of tourist destinations are recommended to increase the resources allocated to the design stage of awareness campaigns, incorporating original and creative graphic designs that at the same time incorporate elements perceived as familiar to the public. The present study represents a benchmark that can guide the managers of tourist destinations in the process of creating and designing future awareness campaigns aimed at promoting sustainability and responsible behaviours towards natural resources and local communities. Therefore, based on the knowledge acquired through this study, a graphic and conceptual design that incorporates the following characteristics is recommended:

- Signs and typography must be simple and easy to identify and recognize.
- Textual messages must be clear and concise.
- The colours must make the text and signs stand out in relation to the rest of the elements and must help to correctly distinguish between them the different graphic elements that make up the composition.
- The composition and distribution of the graphic elements must be orderly and efficient in the use of space.
- It is necessary to incorporate elements that are familiar and known to the public, and/or that attract attention due to their originality.
- The design must be adaptable to different formats of printed and digital documents, as well as to different measures and proportions, to allow their correct dissemination through different channels (television, radio, press, signage, social networks, billboards, etc.) and adapt it to the needs of the different collaborators and intermediaries involved in the dissemination.

Although this study found that it can be very beneficial for tourist destination managers to apply graphic design strategies in awareness campaigns, it should be noted that other factors also influence the success of awareness campaigns and they must be considered. The ability of people to take on the changes proposed by the awareness campaign also determines the ability for the campaign to achieve its objectives [33]. Although the campaign information is attractive, interesting, clear, and available to tourists and residents, the following measures are proposed to be applied to facilitate the change in behaviour of tourists and residents in tourist destinations with similar needs as the PNRPC:

- The posting of signs on the fences of pasture meadows that contain messages reminding to keep gates closed.
- The installation of bins and containers in car parks and rest areas of the most crowded routes and areas.
- The improvement, repair, and maintenance of the signage for routes and mountain paths to make it easier to follow the paths and prevent people from straying off them.
- The installation of speed bumps on road sections that pass through areas particularly sensitive to noise pollution and wildlife collision.
- Promote alternative routes and mountain paths that avoid areas of great pastoral activity, areas of conflict in the sharing of uses of the space, and/or areas of special natural interest with threatened species of flora and fauna.

### 5.3. Limitations and Future Work

It was possible to collect 149 valid responses from the survey. The sample size was sufficient to perform the PLS-SEM analysis with positive indicators of validity and statistical reliability, and the hypotheses of the proposed conceptual model were correctly validated. However, the data collected do not follow a normal distribution, which forced the use of non-parametric statistical analysis techniques, which provide less reliable statistical estimates than parametric techniques, even though they are less sensitive to aspects such as the size of the sample, the distribution of the data or the lack of some of data [52]. Therefore, future research is recommended to work with larger samples to better fit a normal distribution, opening the possibility to use parametric techniques.

In addition, the simplification and reduction of the number of questions recommended by the PNRPC administration meant that each construct is represented by a small number of indicators, making the estimation of the latent variables and the correlations more susceptible to the error factors of the observable variables, thus affecting the representativeness and replicability of the analysis. It is recommended that future studies increase the number of questions per construct in order to counteract that limitation.

Finally, the conceptual model presented in this study can be adapted for research that seeks to understand the role of graphic design and semiotics in advertising campaigns aimed at promoting tourist destinations, tourist products and services, or even destination brands. Meanwhile, the influence of the graphic design’s semiotics of communication and advertising campaigns on tourist destination images remains unexplored [19], and this is where this study can become a reference for future research.

## Figures and Tables

**Table 1:** Sample of related literature.

Reference	Main Topic	Related Content
Barnard (2005) [18]	Graphic design	Graphic design and communication
Lazard (2015) [20]	Graphic design	Putting environmental infographics centre stage
Landa (2018) [6]	Graphic design	Graphic design solutions
Liang (2021) [30]	Graphic design	Information graphic design in visual communication design
Culler (1981) [16]	Semiotics	Semiotics of tourism
Echtner (1999) [15]	Semiotics	The semiotic paradigm: Implications for tourism research
Peverini (2014) [23]	Semiotics	Environmental issues in advertising: A semiotic perspective
Restrepo (2014) [32]	Semiotics	Graphic design production as a sign
Catellani (2022) [17]	Semiotics	Semiotic analysis of environmental communication campaigns
Brakus et al. (2009) [24]	Branding	Sensory, affective, and behavioural brand experience
Adamus (2021) [14]	Branding	Visual component of destination brands
Shukla et al. (2023) [10]	Branding	Sensory, affective, and cognitive brand experience
Maibach (1993) [21]	Awareness	Information campaigns to promote environmental awareness
Macharia et al. (2010) [34]	Awareness	The importance of awareness in biodiversity conservation
Borawska (2017) [5]	Awareness	Public awareness campaigns for sustainable development
Arnberger et al. (2012) [25]	Area preservation	Related factors: protection of nature and guidance of visitors
Budeanu (2007) [33]	Sustainable tourism	Sustainable tourist behaviour
Blancas et al. (2015) [12]	Sustainable tourism	European sustainable tourism labels
Cristobal et al. (2020) [1]	Sustainable tourism	Sustainable tourism marketing
Holmes et al. (2021) [36]	Sustainable tourism	Pro-ecological behaviour at home and abroad

**Table 2:** Variables from the conceptual model (Figure 1) considered for the SEM analysis.

Constructs—Latent Variables (LV)	Code of LV	Questions—Observable Variables (OV)	Code of OV
Semiotics of the design of the awareness campaign [10]	Sem	Colours, images and icons help to understand and identify the messages and concepts of each of the visual creations	V-S1
		Colours, images and icons draw attention to the campaign	V-S2
		The campaign design as collectable badges/medallions, draws attention and encourages the reading of the messages	V-S3
		The use of well-known French songs draws attention to the campaign	V-S4
		The predominance of the colour green reflects the brand of the park, the values and the objective of the campaign	V-S5
Experience (sensory, emotional, and cognitive) by the design of the awareness campaign [10]	Exp	You like the visual design of the campaign	V-E1
		The campaign has awakened emotions (positive and/or negative) in you	V-E2
		The campaign has made you reflect on the topics covered by the campaign and your behaviour	V-E3
Awareness: Changes in thinking and behaviour of the audience from the awareness campaign [24]	Awa	After experiencing the campaign, you paid –or will pay– more attention to the preservation of nature and pastoral activities during your stay in the park	V-A
Preservation of the destination (Park natural resources) [25]	Pre	Since the beginning of the campaign (2021), you have noticed improvements related to the preservation of nature and pastoral activities of the park	V-P

**Table 3:** Characteristics of respondents (*N* = 149).

Question	Category	*N*	%	Mode
Profile	Inhabitant	96	64.43	Inhabitant
	Regular tourist	41	27.52	
	Punctual tourist	12	8.05	
Understanding of written French	Yes	149	100.00	Yes
	No	0	0.00	
Age	0–18	0	0.00	35–60
	19–34	23	15.44	
	35–60	79	53.02	
	61–99+	47	31.54	
Gender	Man	55	36.91	Woman
	Women	92	61.74	
	Other	2	1.34	

**Table 4:** Construct reliability and validity, plus variance R2 and predictive relevance Q2.

Latent Variables	Cronbach’s Alpha	Composite Reliability	AVE	R2	Q2
Awareness (endogenous)	1.000	1.000	1.000	0.491	0.477
Experience (endogenous)	0.838	0.902	0.754	0.699	0.513
Preservation (endogenous)	1.000	1.000	1.000	0.424	0.406
Semiotics (exogenous)	0.898	0.925	0.713		

Note. AVE: average variance extracted.

**Table 5:** Cross loading criterion.

	V-A	V-E1	V-E2	V-E3	V-P	V-S1	V-S2	V-S3	V-S4	V-S5
Awa	1.000	0.491	0.487	0.817	0.597	0.472	0.440	0.544	0.370	0.445
Exp	0.701	0.890	0.861	0.854	0.520	0.765	0.762	0.727	0.607	0.656
Pre	0.597	0.402	0.427	0.518	1.000	0.452	0.455	0.505	0.431	0.440
Sem	0.540	0.837	0.681	0.659	0.541	0.894	0.906	0.874	0.731	0.805

**Table 6:** Correlation between latent variables, and testing of hypotheses (overall data).

Hip.	Correlation	Path Coef.	t-Stats	*p*-Value	Confidence Interval	Conclusion
H1	Sem → Exp	0.836	25.606	0.000	0.764–0.890	Supported
H2	Sem → Pre	0.308	4.266	0.000	0.175–0.455	Supported
H3	Exp → Awa	0.701	12.297	0.000	0.576–0.799	Supported
H4	Awa → Pre	0.430	5.368	0.000	0.263–0.576	Supported

Note. Complete bootstrapping: subsamples = 5000; significance level = 0.05 (two tailed).

**Table 7:** Path coefficients (segmented data).

Hip.	Correlation	Inhabitant	Tourist	Man	Woman	Age > 60	Age < = 60
H1	Sem → Exp	0.854	0.783	0.878	0.771	0.770	0.860
H2	Sem → Pre	0.275	0.306	0.314	0.256	* 0.202	0.362
H3	Exp → Awa	0.731	0.652	0.699	0.694	0.573	0.761
H4	Awa → Pre	0.503	0.353	0.444	0.427	0.493	0.391

Note. Complete bootstrapping: subsamples = 5000; significance level = 0.05 (two tailed); All coefficients have a significance level *p* < 0.05, except * *p* > 0.05.

## Data Availability

Data is unavailable due to privacy restrictions.
